# A Practical Tool for the Assessment of Polymer Biodegradability in Marine Environments Guides the Development of Truly Biodegradable Plastics

**DOI:** 10.3390/polym15040974

**Published:** 2023-02-16

**Authors:** Ricardo Beiras, Sara López-Ibáñez

**Affiliations:** 1 Centro de Investigación Mariña ECIMAT-CIM, Universidade de Vigo, 36331 Vigo, Galicia, Spain; 2 Facultade de Ciencias do Mar, Universidade de Vigo, 36310 Vigo, Galicia, Spain

**Keywords:** biodegradation, biological oxygen demand, biopolymer, marine pollution

## Abstract

Environmental persistence is one of the few shortcomings of plastic materials. As a consequence, alternative plastics labeled as compostable are replacing polyolefins in some commercial applications, such as food bags and trash bags. A rapid, high-throughput, and environmentally relevant method to assess the potential biodegradability in marine conditions is used to assess these materials already on the market, as well as novel bio-based polymers still in development. By fitting experimental data to a non-linear logistic model, ultimate biodegradability can be calculated without regard for incubation time. Whereas the commercial products show negligible or very low marine biodegradability, one of the novel materials exceeds the 20% biodegradation threshold relative to fully marine biodegradable PHB after 28 days. In addition, the sensitivity of the method can be enhanced and its duration reduced, at the expense of labor-demanding preconditioning of the microbial inoculum, by increasing the bacterial density in the incubation vessels. In contrast, pre-exposure of the inoculum to plastic, either in laboratory or field conditions, does not enhance the performance of the test.

## 1. Introduction

Environmental persistence is one of the few shortcomings of conventional plastic materials. Because of that, intentional or unintended disposal of plastic items in the aquatic environment causes not only aesthetic nuisance in landscapes but also the risk of entanglement, smothering, and obstruction of the digestive system in wildlife [1]. 

In an attempt to solve these environmental problems, political initiatives such as the recent European Plastics Strategy [2] prompted the use of natural substances (biomass) as raw materials for the production of plastic products, so-called ‘bio-based’ polymers, and the search for alternative polymers that are biodegradable, at least under industrial composting conditions. The terms ‘bioplastic’ and ‘biopolymer’ are loosely used sometimes to mean biodegradable, i.e., amenable to mineralization leading to CO_2_ and H_2_O in aerobic conditions [3], and other times simply to indicate that they are synthesized from biomass, which can be the case also for conventional non-biodegradable polymers such as polyethylene (PE) or PET. Moreover, some bio-based polymers (e.g., polylactic acid, PLA) are hardly biodegradable in environmentally relevant conditions, while some polymers synthesized using monomers obtained from oil and natural gas (e.g., polycaprolactone) are readily biodegradable [4]. There is thus a need to develop environmentally relevant protocols to test actual biodegradability in environmental compartments and restrict the use of commercial labels that may be misleading in order to avoid greenwashing procedures and provide the customer with technically sound information useful for proper end-of-life disposal.

Biodegradability in aerobic conditions can be assessed by O_2_ consumption or CO_2_ evolution [5]. Alternative polymers replacing polyolefins, including oil-based polybutylene-adipate-terephthalate (PBAT) [6] and bio-sourced PLA, are amenable to mineralization by soil microorganisms in composting facilities [7]. PBAT presents a remarkable performance in terms of processability [8], and it is the main component of current compostable plastic bags [9]. However, biodegradability is a property associated with a given environmental compartment, and these novel commercial materials show very limited biodegradability in aquatic habitats [10,11]. Conversely, polyhydroxyalkanoates (PHAs), such as poly-3-hydroxybutyrate (PHB) or poly(3-hydroxybutyrate-co-3-hydroxyvalerate) (PHBV), have been tested in diverse environmental conditions, including seawater (scarcely explored for this type of testing compared to soil and other environments), showing high biodegradation rates [12]. The potential of PHAs has been greatly acknowledged, and they are being produced by several companies around the world, although this material still requires high production costs and shows a significant fragility when processed with conventional techniques [13]. A comprehensive assessment scheme for plastic products, including packaging and coatings, designed to be biodegradable under aerobic marine conditions was issued by ASTM in 2005 but withdrawn in 2014. This scheme [14] included three types of criteria: mechanical disintegration (70% of the material after 84 days assessed by 2 mm sieving), biodegradation (at least 30% assessed by CO_2_ evolution within 180 days, among other requirements), and lack of ecotoxicity in four toxicity tests using microbes, algae, daphnia, and fish. Biodegradation was assessed according to ASTM D6691 after very long incubations (180 days) at environmentally irrelevant conditions (30 °C) [15]. Subsequent developments intended to standardize plastic biodegradation tests in different marine habitats [16,17,18,19,20,21,22] continue to propose extremely long incubation periods (from 120 days to 2 years) that greatly limit the throughput of the technique, increase costs, and enhance the likelihood of accidental loss of data during the testing period. Recently, we developed a rapid protocol to test marine biodegradability of polymers that allows substantial reduction of exposure time and thus increases throughput capacity by formulating a nutrient composition representative of marine conditions, using a microbial inoculum directly obtained from marine sediments, and pretreating the tested material to particle size reduction down to <250 µm [11].

Within the EU-funded Glaukos Project, novel polymers are being developed from bio-based feedstocks. In this study, a recently developed rapid method to test marine biodegradability was used to compare the performance of these novel materials with some commercial compostable bags and other alternative plastics currently available on the market. In addition, the effects of both inoculum preconditioning and pre-exposure as defined by ISO 16221 were also assessed. Preconditioning is the previous incubation of the inoculum under the conditions of the subsequent test with the aim of improving its performance by acclimatization of the microorganisms, whereas pre-exposure is the previous incubation of the inoculum in the same kind of material to be tested to select microorganisms with the ability to degrade the test material [23].

## 2. Materials and Methods

### 2.1. General Methods

Aerobic biodegradation of polymeric materials in marine water was assessed according to the method developed by López-Ibáñez and Beiras [11]. Briefly, 100 mg/L test samples were incubated in 0.5 L amber glass bottles at 20 ± 0.1 °C in the dark for 28 days, and the Biological Oxygen Demand (BOD) was recorded daily by means of OxiTop^®^ (WTW, Weilheim, Germany) pressure sensor caps and the use of KOH pellets to capture the evolved CO_2_. The incubation media consisted of 0.8 µm filtered seawater sterilized with UV light and enriched with N and P according to the Redfield ratio [24]. NO_3_ rather than ammonium was used as the N source to better mimic marine natural conditions and limit the potential interference of the nitrogenous oxygen demand that may otherwise overestimate carbonaceous BOD [25]. A marine liquid inoculum of sediment pore water (SPW) (1% of the water volume) was added to each bottle, including blanks. The SPW was obtained by digging ca. 30 cm deep into the sand during low tide at a small beach located in an urban area (42°11′49.7″ N 8°47′45.2″ W) and collecting the water in the hole with a sterile glass bottle. This liquid inoculum was used immediately after collection. Headspace was allowed in the bottles in order to increase the available O_2_. Each trial included blanks, positive controls (C+) of PHB resin powder (ID019, Table A1), and the test samples, with two replicates each. For each trial, the mean BOD value of the blanks is subtracted from the BOD of the treatments. The resulting blank-corrected BOD values are expressed as a percentage of the BOD recorded in the positive control.

### 2.2. Effects of Inoculum Preconditioning and Pre-Exposure

Some authors [26] advocate a previous step of preconditioning or “boost” intended to increase bacterial density in the inoculum in order to achieve more robust results. This inoculum preconditioning can also shorten the lag phase and thus the test duration. Therefore, we performed experiments using a home-compostable commercial bag and a pelletized PHB resin (ID016 and ID020 in Table A1), increasing the bacterial density in the inoculum by pre-incubation in marine broth. With that aim, 100 mL of the basic SPW marine inoculum (in triplicate) was filtered using sterilized 0.22 µm glass-fiber filters and incubated in 250 mL Erlenmeyer flasks with marine broth (PanReac AppliChem, 40 g L^−1^). Flasks were incubated in an orbital shaker (150 rpm) for 72 h at 20 °C in darkness, and the number of viable bacteria per mL, termed in microbiology colony-forming units (CFU), were counted on marine agar plates (72 h, 20 °C, darkness). This allowed calculation of the volume of preconditioned inoculum needed to achieve a final concentration in the BOD bottles of 10^5^ (as recommended by international standards [23]) and 10^7^ CFU mL^−1^. The fresh inoculum was also plated after collection and incubated as described above in order to count the CFU mL^−1^ in the bottles seeded with fresh (not preconditioned) inoculum.

Another method that is potentially useful to increase the efficiency of the test is to pre-expose the inoculum used to the materials to be tested, with a view to selecting those microorganisms capable of using those materials as a carbon source. With that aim, we compared the use of the previously studied SPW marine inoculum with two other sources of microorganisms: weathered plastic ropes stranded on a beach and perlon wool from an aquarium filter unit. In both cases, those microbial communities had been pre-exposed to plastics, in the first case in natural conditions and in the second case in aquariums where 100 mg L^−1^ of PHB powder (ID019, Table A1) was periodically added. 

For obtaining the pre-exposed inoculum from the different materials, they were cut down to small pieces, inserted in 10 mL tubes with sterile seawater, and vortexed for two minutes. Then, the liquid medium was extracted and used to seed the BOD bottles.

### 2.3. Effect of Particle Size and Shape

We tested the effect of material presentation (film, pellets, and powder) and particle size on biodegradation rates. We used PHB pellets, PHBV pellets, and a compostable bag as testing materials (ID020, ID022, and ID016, respectively, Table A1), with the following presentations: three mm diameter pellets (ID020 and ID022), one cm2 film pieces (ca. twenty µm) from ID016, and powder obtained from the three materials after micronization and sieving by 1 mm and 250 µm metallic meshes.

### 2.4. Application to Commercial and Customized Plastics

We studied the marine biodegradability of the 1st and 2nd generation customized polymers and coatings provided by the GLAUKOS project and commercial plastic products, including compostable materials and a conventional PE bag used as a negative control (Table A1). Except where otherwise stated, materials were previously micronized using a ZM200 ultracentrifuge mill (Retsch, Verder Scientific, Haan, Germany and sieved through a 250 µm metallic mesh. For commercial products, CACTI central services (University of Vigo) identified the base polymer by Fourier-Transform Infrared spectroscopy (FTIR) using a Thermo Scientific Nicolet 6700 (see Table A1). 

### 2.5. Quality Assurance

The BOD_28_ in the blanks was always less than 2% of the positive control. Low blank BOD values translate to high signal-to-noise ratios that enhance the power of the method to discriminate between materials with different degrees of biodegradability. The BOD_28_ in the positive controls was always >60% Theoretical Oxygen Demand (ThOD), meeting the standard acceptability criteria [20,27].

Although the short incubation times and use of NO_3_^-^ rather than NH_4_^+^ as a N source makes significant nitrification activity unlikely, the potential overestimation of the BOD due to oxidation of inorganic nitrogen during incubation was quantified by running additional replicates of the reference material PHB (ID019) with 6 mg/L of the nitrification inhibitor allyl-thio-urea. The nitrification-inhibited treatment reached 91.9% BOD_28_ compared to the control, C+.

### 2.6. Assessment Criteria for the Classification of Marine Biodegradability

Regarding assessment criteria, 60% of ThOD has been frequently invoked as a threshold for ready biodegradability [23,27,28,29], and 20% of ThOD is used by [30] as a pre-screening criterion to consider a material as potentially biodegradable in the marine environment. However, during microbial biodegradation, a relevant proportion of the polymer carbon is not mineralized to CO_2_ but assimilated by the heterotrophic microbial consortium and converted into biomass, setting an actual maximum of BOD between 30 and 50% below ThOD [31]. On the other hand, current biodegradable materials are frequently heteropolymers and complex mixtures whose exact atomic composition is unknown, which prevents calculation of the ThOD. Because of these limitations, we have recently proposed replacing the percentage of ThOD by the percentage of the BOD recorded in the positive control (C+), using as C+ the truly marine-biodegradable polymer PHB [11]. 

In addition, since the current method is based on short-term (28 days) incubations, it is advisable to include a third benchmark intended to differentiate fully non-biodegradable materials from slightly biodegradable materials, which can be arbitrarily set at 5% C+. Therefore, a provisional scheme for the assessment of marine biodegradability of plastic materials can be based on these benchmarks, resulting in the following classes (Table 1): (i) non-biodegradable (<5% C+ in 28 days), (ii) slightly biodegradable (between 5 and 20%), (iii) moderately biodegradable (between 20 and 60%), and (iv) readily biodegradable (>60%) (see Table 1).

### 2.7. Statistical Methods

The BOD values recorded during the 28-day incubations were fit into a logistic regression model according to the equation:

(1) $$Y=\frac{BOD_{L}}{1+10^{\left(Log\;a-Log\;X\right)*b}}$$

where *Y* is BOD (mg L^−1^), *X* is time (days), *a* is the half-degradation time, and *BOD_L_* is the ultimate or limit BOD. This model provides two parameters, the slope (*b*) and the asymptote (*BOD_L_*), which allow you to estimate, respectively, the biodegradation rate and overall biodegradability of a given material independent of exposure time, provided the data show a good fit to the mathematical model used. The suitability of this model to fit experimental data was compared with two alternative models: fixed slope curves, where *b* = 1:

(2) $$Y=\frac{BOD_{L}}{1+10^{\left(Log\;a-Log\;X\right)}}$$

and asymmetric curves, where an additional parameter, *S*, is introduced, accounting for the degree of asymmetry:

(3) $$Y=\frac{BOD_{L}}{[1+\left(2{\;}^{\frac{1}{S-1}}\right)*{\left(a/X{)}^{b}\right]}^{S}}$$

Model comparisons were conducted according to Motulsky [32], running the F-test and using the Akaike Information Criterion (AIC). In the former, it is assumed that a low *p*-value corresponds to a better fit of the most complex model (the one including more parameters), whereas for the latter, a positive or negative value indicates the preferable model: the most complex or the simplest one, respectively. IBM SPSS (version 25) and GraphPad Prism (version 8) were used for the statistical analyses. 

## 3. Results and Discussion

### 3.1. Effect of Inoculum Preconditioning and Pre-Exposure

**Figure 1:**
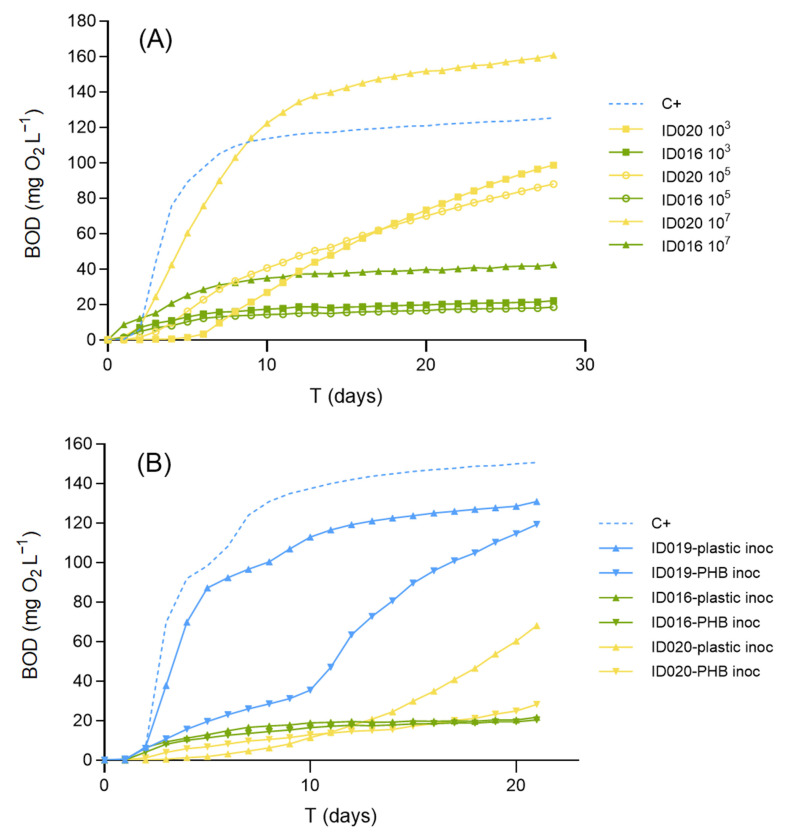
Effects of inoculum preconditioning (**A**) and pre-exposure (**B**). In (**A**), the performance of the marine inoculum dosed as prescribed in standard methods (10^3^) is compared with two preconditioned treatments previously incubated to enhance bacterial density to 10^5^ (circles) and 10^7^ (triangles) CFU mL^−1^ with the materials ID016 (home compostable bag) and micronized ID020 (PHB pellets). Notice that the latter treatment remarkably increased the biodegradation of both materials. C+ is also tested at 10^3^ UFC mL^−1^. In (**B**), the standard inoculum, taken from sediment pore-water, is compared with two pre-exposed inocula: one taken from naturally weathered plastics from a beach (“plastic inoc”), and another obtained from the filter of an aquarium where PHB powder was periodically dosed (“PHB inoc”), using as test materials the PHB powder (ID019) and both ID016 and ID020 again.

Using the fresh marine inoculum in the quantities prescribed in standard BOD methods results in a bacterial density in the incubation medium of ca. 10^3^ CFU mL^−1^. Preconditioning the inoculum in order to increase the bacterial density in the bottles up to 10^5^ CFU mL^−1^ failed to affect biodegradation rates (Figure 1a). However, the 10^7^ CFU mL^−1^ treatment showed remarkably higher BOD values for both the home-compostable bag (ID016) and the pelletized PHB (ID020) compared to the treatments with standard inoculum (Figure 1a). BOD_28_ increased from 22 to 42 mg/L for the former and from 99 to 161 mg L^−1^ for the latter. Therefore, a previous step of inoculum preconditioning to achieve 10^7^ CFU mL^−1^ may slightly improve the sensitivity of the test. However, preconditioning mainly seems to reduce the lag phase of the microorganisms’ growth and the BOD_L_ values, especially for ID020 at 10^5^ and 10^7^ CFU mL^−1^, which are not very different. Moreover, preconditioning makes the method more labor-intensive since it demands conducting agar plate and liquid marine broth incubations for each run of BOD bottles tested. 

When the performance of the basic marine inoculum was compared with the two other inocula, pre-exposed to either natural plastics (inoculum obtained from beach-weathered plastics) or specifically to PHB (inoculum obtained from an aquarium where PHB was periodically added), pre-exposure did not improve the results (Figure 1b). BOD_21_ expressed as %C+ for the home-compostable bag was 16.6%, 16.5%, and 17.0% for the basic, beach plastic pre-exposed, and PHB pre-exposed inocula, respectively, whereas for the pelletized PHB, values were 63.2%, 52.0%, and 23.7%, respectively. Moreover, when the purpose of the test is to represent natural environmental conditions, the standard methods recommend avoiding pre-exposure of the inoculum to the testing materials [29]. Nevertheless, the inoculum extracted from environmental plastics performed better than the artificially selected inoculum from the PHB-dosed aquarium. This could be due to the higher diversity of microorganisms present in the natural plastics collected from the beach.

### 3.2. Use of the Logistic Model to Estimate Biodegradability

The three-parameter logistic model (Equation (1)) allows modeling BOD (mg L^−1^) as a function of time (days) and estimating three parameters that describe the mineralization kinetics: the time corresponding to half degradation (*a*), the slope (*b*), and the asymptote of the curve (BOD_L_). Provided experimental data show a good fit, the model allows objective quantification of two important properties of each material: the rate or speed of biodegradation (assessed by *b*) and the ultimate biodegradability of a given material with independence of exposure time, assessed by BOD_L_. In order to compare the goodness of fit of this variable slope model (Equation (1)) versus the alternative logistic models of fixed slope (Equation (2)) and asymmetric (Equation (3)), the experimental data for materials ranging from 100 down to 5% degradation were used.

As summarized in Table A2, in all cases, the variable slope model (Equation (1)) was preferred to the fixed slope model (Equation (2)) to fit the experimental data. The introduction of an asymmetry coefficient slightly improved fitting for the three most biodegradable materials (*p* < 0.05 and positive AICs). However, in the cases of the two least degradable materials, the variable slope model was preferred according to the Akaike Information Criterion and a value of *p* > 0.1 did not support the asymmetric model. In addition, even when a significant improvement in fitting was identified, the value of *S* (degree of asymmetry) could not be confidently estimated for any of the materials tested, as indicated by the 95% confidence intervals spanning from minus infinite to plus infinite (data not shown). Consequently, the variable slope model was chosen to estimate BOD_L_ values.

### 3.3. Effect of Particle Size and Shape

**Figure 2:**
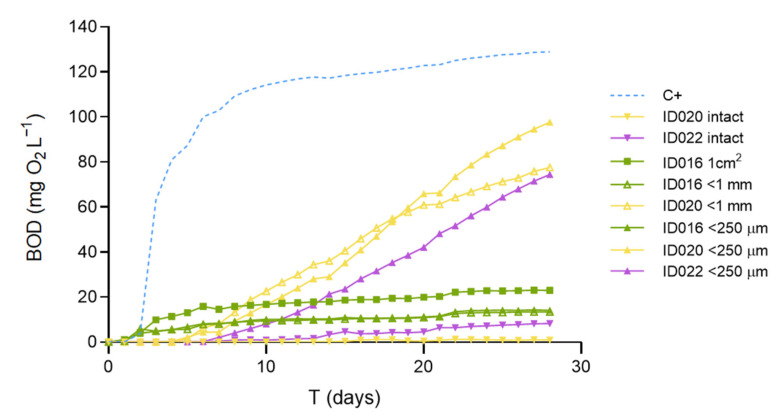
Effect of particle size and shape on the marine biodegradation of PHB (ID020) and PHBV (ID022) pellets and a home-compostable bag (ID015). The resins consisted of 3 mm pellets that were tested intact and after micronization down to <1 mm and <250 µm. The bag was tested, cut into 1 cm^2^ pieces, and micronized. C+ is the positive control of PHB powder.

In our previous study, we had shown that micronization highly increased the biodegradability of PHB pellets [11]. As we can see in Figure 2, in the present study, PHB pellet samples ground to either <250 µm or <1 mm showed similarly improved biodegradability compared to intact pellets. Similarly, the PHBV samples did not biodegrade intact over the 28 days of the test but showed remarkable biodegradability after micronization.

**Figure A1:**
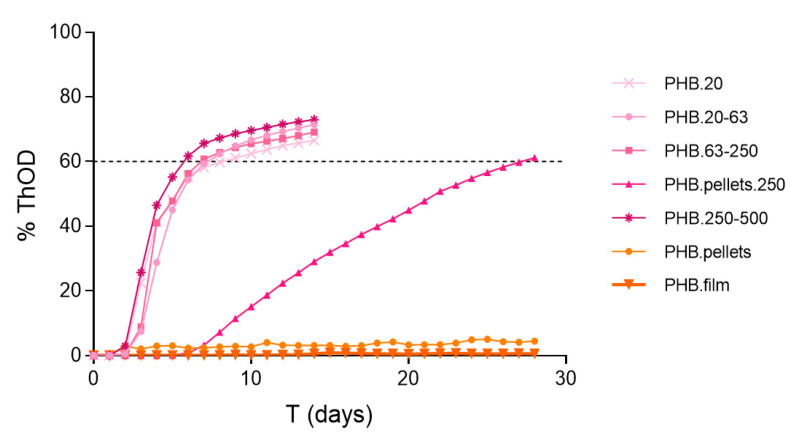
Percentage of biodegradation expressed as % ThOD for PHB particles with size ranges of: ≤20 µm, 20–63 µm, 63–250 µm, and 250–500 µm, pellets of 1–3 mm, micronized pellets <250 µm, and 100 µm thick films. Data for PHB pellets and film taken from [11].

The home-compostable bags tested showed practically identical results, disregarding the degree of micronization. Moreover, this material, which is presented as very thin films (ca. 20 µm thick), yields similar results even when tested in the form of 1 cm^2^ pieces without previous grinding. In the three cases, the BOD_28_ expressed as a percentage of the positive control ranges from 10.9 to 17.8%, which provides a consistent classification as slightly biodegradable in marine conditions (see Table 1). Additional trials conducted with the positive control material (PHB powder) sieved to separate different particle size fractions (Figure A1) reinforce the conclusion that, provided a minimum weight-specific surface area is present, the biodegradability results obtained with the present methodology are consistent and independent of the material’s particle size. Therefore, the present results support the notion that grinding is a requirement for short-term assessment of biodegradability in pelletized materials. In the case of thin films (ca. 20 µm), weight-specific surface area may be sufficiently high for microbiota to promote mineralization in the short time interval of the current method (28 days). Nevertheless, that may not apply to all polymers but only to those susceptible to being degraded by the naturally present microorganisms without the need for previous abiotic degradation. Therefore, we suggest micronizing all types of materials when searching for potential biodegradability, as well as further testing with different types of thin films. 

By fitting the experimental data to a logistic model (Equation (1)), the ultimate BOD (BOD_L_) can be estimated. This parameter provides an assessment of the material’s biodegradability that is more independent of the specific biodegradation kinetics performed in the incubation vessels than the BOD_28_. This can be illustrated by comparing the slopes and BOD_L_ values from Equation (1) for the reference materials tested at different particle sizes (Table A3). Notice that ground PHB pellets show a significantly slower biodegradation rate (*b*) and thus a lower BOD_28_ value but the same ultimate biodegradability (BOD_L_) as for the PHB powder stock, maybe due to the presence of different polymer additives employed for pelletization [11].

### 3.4. Application to Commercial and Customized Plastics

**Figure 3:**
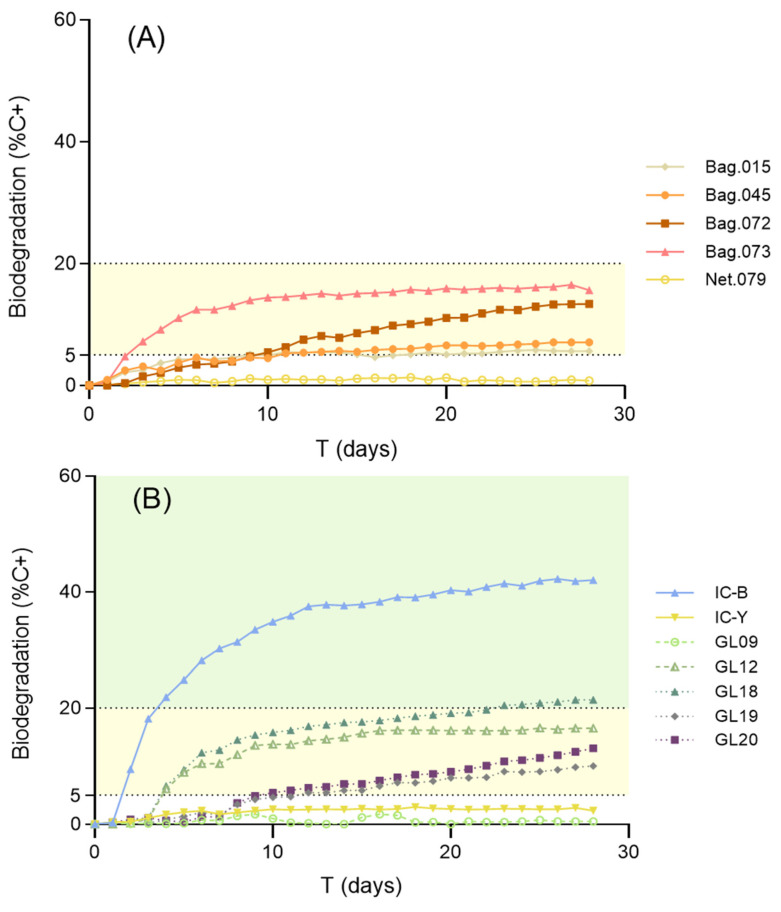
Results of the standard 28 day marine biodegradability test for (**A**) commercial compostable bags and (**B**) Glaukos Project first-generation polymers (GL09 and GL12), 2nd-generation polymers (GL18, GL19, and GL20), and coatings (IC-Y and IC-B). BOD data are expressed as a percentage of the reference material, PHB (%C+). All products micronized to ≤250 µm. Slight and moderate biodegradability are marked as yellow and green shades, respectively. Notice that the alternative coating IC-B shows remarkable marine biodegradability, and the second-generation polymer GL18 outperforms all commercial materials.

Table 2 summarizes the classification of the tested materials according to their marine biodegradability. In a previous study using the same methods, we had shown the limited biodegradation of compostable plastic bags in marine conditions. The present study tested additional compostable plastic materials and confirmed their poor performance in terms of marine biodegradability (Figure 3a). None of these materials reach the 20% biodegradation threshold needed to be considered at least moderately biodegradable in marine conditions. The most biodegradable compostable bag found (ID073) reached a value of 15% C+ after 13 days of exposure but showed a plateau during the last two weeks of exposure. This biodegradation kinetics suggests the presence of some easily leached, soluble, and/or highly biodegradable component in the composition of this material rather than actual mineralization of the main polymeric matrix. Moreover, one of the compostable materials (ID079) showed a result very similar to the negative control (conventional polyethylene).

Concerning customized materials, the second-generation polymer GL18, supplied by the Glaukos Project, showed a value of 21.4% degradation compared to C+. The oxygen-demand kinetics of this material show progressive degradation with no plateau and support actual mineralization of the polymeric matrix. The Glaukos Project is currently in progress to further improve the biodegradation rates without decreasing the mechanical performance of these novel bio-based materials. 

Many marine applications (e.g., nets) demand the use of coatings to improve the performance of plastic materials. The alternative coating supplied by I-Coats (IC-B) reached a marine biodegradation rate of 42% in 28 days, far beyond the value obtained for a conventional coating (IC-Y) (2.3%).

The hydrolysable ester bonds of PHB and other poly-hydroxy-alkanoates enable relatively rapid degradation of these polymers in environmentally relevant conditions representative of terrestrial [33] and aquatic habitats [11,34]. PHB-degrading bacteria have been isolated from marine sediments [35,36] and from the water column [37]. Unfortunately, due to its mechanical properties, PHB currently has limited commercial value [38]. Although it has been studied for some applications, like packaging [39], it is not easy to find in the carrier bag market, trash bags, or similar items made only from this polymer. It normally appears blended with other substances that improve the final properties of the product [8], and the final biodegradability of the blend may be very different from the original PHB biodegradability. PBAT, although oil-based [6], presents a better performance in terms of processability [8] and is the main component of current compostable plastic bags. However, both the PBAT resin and PBAT-based bags have shown very limited marine biodegradability ([11] in the present study). PLA is another alternative polymer used for different applications that, similar to PHB, requires high production costs. Even so, it is in the spotlight due to its sustainable source, potential biodegradability, and attempts to enhance its physical and mechanical properties in order to implement its use for household items and packaging [40,41]. In previous experiments, it has achieved negligible biodegradation rates in both forms (resin and final product) under marine conditions, although this increases when blended with other polymers [11]. In contrast, some of the novel bio-based polymers and alternative coatings tested in the present study have shown remarkable marine biodegradation (see Figure 3) and the potential to be used for applications where the risk of being lost in the sea is mitigated by their biodegradable nature.

## 4. Conclusions

Because of its short duration (28 days) and environmental relevancy, the method presented here is useful to classify commercial plastic products according to their potential biodegradability in marine conditions and to guide the development of novel polymeric materials intended to rapidly degrade in the sea.

In most trials, the experimental data showed a good fit to a non-linear logistic model. This allows estimation of the BOD_L_ from the asymptote of the curve. This parameter assesses ultimate biodegradability in a more robust and kinetics-independent way than BOD_28_.

When very thin (ca. 20 µm) films are assessed, micronization pretreatment is not always necessary but highly recommended, whereas when more compact materials are assessed, micronization to reduce particle size to at least less than 1 mm is essential. Furthermore, it has been proven that, below 250 µm, different fractions behave similarly (Figure A1), not accelerating the biodegradation rate as they decrease in size. Given that, it would be enough to micronize at a maximum of ≤250 µm. 

The test may be further accelerated by increasing the density of marine heterotrophic microorganisms in the incubation vessels up to at least 10^7^ CFU mL^−1^ by means of a previous boost step using marine broth, since it would reduce the lag phase. In contrast, pre-exposure to plastics in both natural and laboratory conditions did not enhance the performance of the inoculum.

The method used in this study allows for the rapid assessment of the potential biodegradability of novel experimental materials in a short period of time, providing rapid feedback to polymer designers and thus accelerating the technological development needed to produce environmentally friendly customized products.

## Figures and Tables

**Table 1:** Proposed assessment criteria for the classification (four-colored) of marine biodegradability of plastic materials according to the % of BOD_28_ obtained for a positive control (C+) consisting of the fully biodegradable polymer PHB.

% C+	Category
>60	Readily biodegradable
20 < x ≤ 60	Moderately biodegradable
5 < x ≤ 20	Slightly biodegradable
≤5	Non-biodegradable

**Table 2:** Classification of the tested materials according to their marine biodegradability using the assessment criteria shown in Table 1, and fitting parameters using a non-linear logistic model (Equation (1) in the text). Ninety-five percent confidence intervals for the parameters are shown in parentheses. PE: polyethylene; n.c.: not calculable.

Material	%C+	Biodegradability	*b*	BOD_L_ (mg L^−1^)
PE bag (ID017)	1.2%	None	n.c.	1.3 (n.c.)
Home-compostable bag (ID016)	17.6%	Slightly	1.40 (1.20, 1.62)	22.2 (21.2, 23.5)
Home-compostable bag (ID045)	7.8%	Slightly	0.85 (0.68, 1.04)	19.8 (16.5, 27.6)
Industrial-compostable bag (ID015)	5.4%	Slightly	1.75 (1.36, 2.23)	7.6 (7.3, 8.0)
Industrial-compostable bag (ID072)	13.4%	Slightly	1.50 (1.32, 1.69)	27.4 (23.7, 33.9)
Industrial-compostable bag (ID073)	15.6%	Slightly	1.84 (1.69, 2.01)	21.6 (21.2, 22.0)
Compostable net (ID079)	0.8%	None	2.81 (0.98, n.c.)	1.2 (1.1, 1.4)
Conventional coating (IC-Y)	2.3%	None	2.21 (1.59, 3.10)	3.5 (3.4, 3.8)
Alternative coating (IC-B)	42.0%	Moderate	1.65 (1.49, 1.83)	54.2 (52.7, 55.9)
GL09	0.5%	None	n.c.	0.1 (n.c.)
GL12	16.5%	Slightly	2.73 (2.33, 3.19)	20.7 (20.1, 21.4)
GL18	21.4%	Moderate	2.24 (1.82, 2.74)	29.2 (27.8, 31.1)
GL19	10.0%	Slightly	1.82 (1.50, 2.18)	18.2 (15.6, 23.2)
GL20	13.1%	Slightly	1.71 (1.25, 2.23)	27.8 (20.5, 64.4)

## Data Availability

The data presented in this study are available on request from the corresponding author.

## Appendix A

**Table A1:** Characteristics of the test materials. PHB: poly-3-hydroxybutyrate; PHBV: poly(3-hydroxybutyrate-co-3-hydroxyvalerate); PE: polyethylene; EVA: ethylene vinyl acetate. For commercial materials, polymer base and other major components were identified by FTIR analysis.

Type	Product Description	Supplier Composition	Source	Polymer Base	Other Components
Reference materials	PHB powder (ID019)	PHB	Helian Polymers	PHB	
	PHB pellets (ID020)	PHB	Helian Polymers	PHB	
	PHBV pellets (ID022)	PHBV	Helian Polymers	PHBV	
Commercial materials	Conventional PE carrier bag (ID017)	PE	Pampols	PE	none
	Home compostable bag-GreenMaker (ID016)	PBAT + PLA + starch	GreenMaker	Polyester	none
	Home compostable bag-Mater Bi (ID045)	MaterBi + starch + biodegradable polymers	BioBag	Polyester	none
	Industrial compostable bag (ID015)	Starch + biodegradable polymers	EcoPack	Polyester	none
	Industrial compostable bag (ID072)	MaterBi + corn starch + plant-based polymers	Saplex	Polyester	Unidentified ester
	Industrial compostable bag (ID073)	Potato starch	Vileda	Polyester	Unidentified ester
	“Bio” net-bag (black) (ID079)	PLA	EcoPlas	Polyester	Talcum
Customized materials	1st generation Glaukos polymers (GL09, GL12)	-	Glaukos	Bio-based poly-condensate	
	2nd generation Glaukos polymers (GL18, GL19, GL20)	-	Glaukos	Bio-based poly-condensate	
	Conventional coating (IC-Y)	-	I-Coats	Polyester-acrylic	none
	Alternative coating (IC-B)	-	I-Coats	Polyurethane	Unidentified ester

## Appendix B

**Table A2:** Comparison of logistic models to fit experimental data for readily biodegradable (PHB powder, C+), moderately biodegradable (bio-based poly-condensate GL18), and slightly biodegradable (compostable bags ID073, ID015, and ID072) materials. AIC: Akaike Information Criterion. For further explanation, see the text.

Comparison	C+	GL18	ID073	ID015	ID072
Fixed slope vs. variable slope					
F (degrees of freedom)	189.7 (1, 25)	50.6 (1, 25)	158.1 (1, 25)	10.3 (1, 25)	35.5 (1, 25)
*p*	<0.0001	<0.0001	<0.0001	0.0036	<0.0001
preferred	Variable slope	Variable slope	Variable slope	Variable slope	Variable slope
Variable slope vs. asymmetric					
F (degrees of freedom)	7.60 (1, 24)	16.17 (1, 24)	14.38 (1, 24)	1.466 (1, 24)	0.7859 (1, 24)
*p*	0.011	0.0005	0.0009	0.2378	0.3841
AIC	4.7	11.4	10.2	−1.33	−2.09
preferred	Asymmetric	Asymmetric	Asymmetric	Variable slope	Variable slope

**Table A3:** Fitting parameters of the BOD curves depicted in Figure A1 to a logistic model (see Equation (1) in Section 2.7). Notice that ground PHB pellets show a significantly slower biodegradation rate (*b*) but the same biodegradability (BOD_L_) as the PHB powder stock.

Material	Treatment	*b*	BOD_L_ (mg L^−1^)
PHB powder (ID019)	sieved <20 µm	0.52 (0.373, 0.754)	62.6 (59.63, 65.61)
PHB powder (ID019)	sieved 20–63 µm	0.47 (0.365, 0.621)	67.5 (64.75, 70.29)
PHB powder (ID019)	sieved 63–250 µm	0.57 (0.398, 0.945)	65.2 (61.91, 68.62)
PHB pellets (ID020)	ground and sieved <250 µm	0.10 (0.085, 0.107)	67.2 (64.57, 70.06)
